# Modeling saccade reaction time in marmosets: the contribution of earlier visual response and variable inhibition

**DOI:** 10.3389/fnsys.2024.1478019

**Published:** 2024-10-23

**Authors:** Wajd Amly, Chih-Yang Chen, Tadashi Isa

**Affiliations:** ^1^Division of Neurobiology and Physiology, Department of Neuroscience, Graduate School of Medicine, Kyoto University, Kyoto, Japan; ^2^Institute for the Advanced Study of Human Biology (WPI-ASHBi), Kyoto University, Kyoto, Japan; ^3^Human Brain Research Center, Graduate School of Medicine, Kyoto University, Kyoto, Japan

**Keywords:** *Callithrix jacchus*, gap saccade task, neural field model, reaction time, inhibition, visual response

## Abstract

Marmosets are expected to serve as a valuable model for studying the primate visuomotor system due to their similar oculomotor behaviors to humans and macaques. Despite these similarities, differences exist; challenges in training marmosets on tasks requiring suppression of unwanted saccades, having consistently shorter, yet more variable saccade reaction times (SRT) compared to humans and macaques. This study investigates whether the short and variable SRT in marmosets is related to differences in visual signal transduction and variability in inhibitory control. We refined a computational SRT model, adjusting parameters to better capture the marmoset SRT distribution in a gap saccade task. Our findings indicate that visual information processing is faster in marmosets, and that saccadic inhibition is more variable compared to other species.

## Introduction

Marmosets are a valuable addition to studying the primate sensorimotor and cognitive functions. Their brain is lissencephalic with less cortical folding, allowing easier access to many brain areas on the exposed cortical surface. They also display rich social behaviors and communication similar to humans, including vocal exchanges, gaze following, social learning, and cooperative breeding (Miller et al., 2016). Furthermore, for early visual areas like the retina, lateral geniculate nucleus (LGN), V1, V2, and middle temporal area (MT), the functional properties and anatomical organization appear broadly similar between marmosets and macaques, aside from eye size differences, making them a promising model for studying active vision and visual cognition (Mitchell et al., 2014; Solomon and Rosa, 2014).

Researchers have shown that marmosets can be trained to perform variety of visual and cognitive tasks, both in head-free and head-restrained conditions, and they exhibit saccadic behavior comparable to that of macaques and humans (Spinelli et al., 2004; Clarke et al., 2011; Mitchell et al., 2014; Johnston et al., 2018; Sedaghat-Nejad et al., 2019; Chen et al., 2021; Jendritza et al., 2021). Despite the similarities in oculomotor behavior that marmosets share with macaques and humans, there are some critical differences, such as the challenge of suppressing unwanted saccades. For instance, it has been described that training marmosets on a blocked antisaccade task is possible only if the task is eased, where they have to generate a saccade away from a dimly lit peripheral stimulus (Johnston et al., 2019). Furthermore, the saccade reaction time (SRT) in marmosets is consistently shorter but often more variable, ranging from 69.7 to 399 ms on average (Ma et al., 2020; Chen et al., 2021).

Understanding the reason behind these differences is a complex issue. However, by modeling various components of neural activity which integrate different types of information that guide saccadic generation in a species-specific manner, we can potentially infer the underlying causes of differential saccadic behavior. We incorporated adjustments to parameters in their model based on our marmoset behavioral data and the cumulative knowledge of marmoset behavior and neurophysiology. Our objective is to refine the model’s parameters to enhance its fidelity in replicating marmoset SRT. This refinement seeks to deepen insights into the factors contributing to the shorter and more variable SRT observed in marmosets compared to humans.

## Materials and methods

### Animal preparation and surgical procedure

Three adult marmosets born in the breeding colony at the Kyoto University Animal Research Facility, aged 3–5 years participated in this study; two males; marmoset J and marmoset P, and one female; marmoset M. The experiments were conducted following the guidelines of the Japan Neuroscience Society and the Science Council of Japan and were approved by the Animal Ethics Committee at Kyoto University, Japan, under license number Med Kyo24059. Marmosets were provided with daily food and water and were not deprived during the experiment.

Following 2 weeks of chair training, marmosets underwent headpost implantation surgery to prepare for head fixation. Each marmoset was aseptically mounted with a custom-designed headpost, tailored to its MRI-base skull reconstruction, under 1.5% isoflurane anesthesia, following induction with 14 mg/kg ketamine and 0.14 mg/kg medetomidine. We used biocompatible Dental SG Resin (Form2, Formlabs, U.S.A.) to 3D print the headpost and attached it to the skull with Super-Bond (Sun Medical Co., Ltd., Japan). This method ensures a stable and long-term fixation.

### Human subjects

Three adult human subjects aged 29–37 years participated in this study, two males; human C and human H, and one female; human W. Approval was obtained from the ethics committees at the Medical Faculty of Kyoto University. All human subjects provided informed, written consent before participating in the experiment. Human subjects did not have their heads fixed; instead, head position was stabilized using a custom-made chin rest.

### Visual task and monitor setup

Stimuli were generated using PsychoPy 3.6 (Peirce et al., 2019). They were displayed on a gray background with a luminance of 99 cd/m^2^ on a Dell AW2521HF LCD monitor for marmosets and 58.47 cd/m^2^ on a Dell AW2125HF LCD monitor for humans, positioned at a distance of 41 cm. Both monitors had a resolution of 1920 × 1080 pixels and operated at a refresh rate of 60 Hz.

**FIGURE 1:**
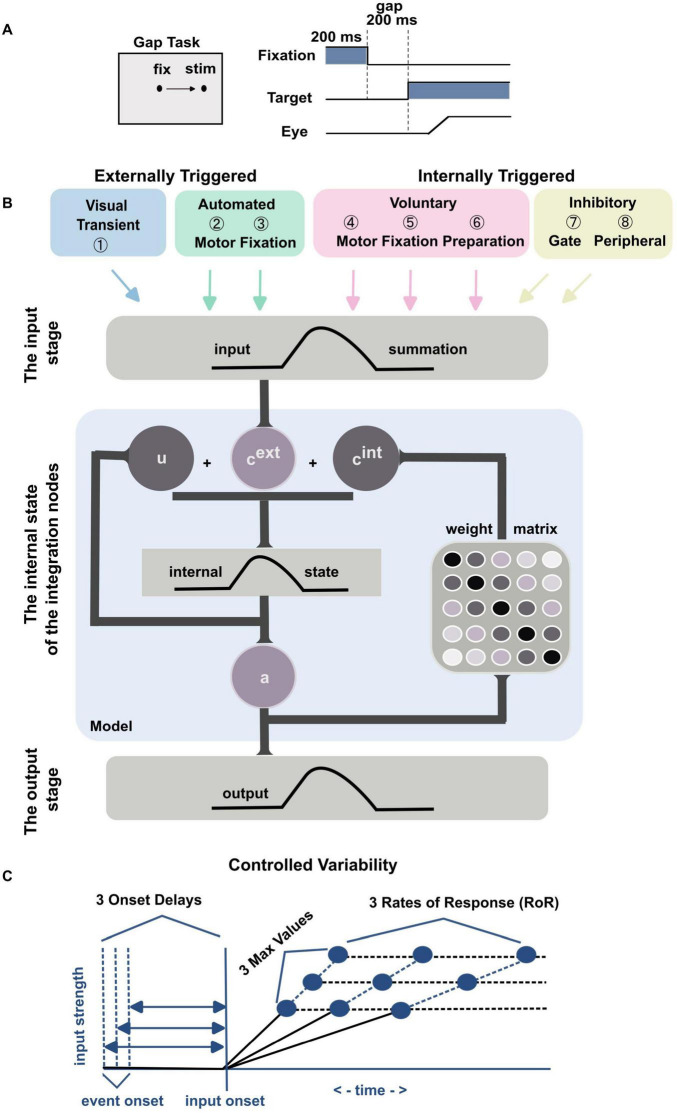
**(A)** Illustration of the visual gap task. Following a 200 ms fixation period, a 200 ms gap period ensued, after which a visual target appeared. Subjects were required to make a saccade toward the visual target. **(B)** Graphical representation of the mathematical model, adopted from Coe et al. (2019), summarize the mathematical model. The initial stage displays the eight inputs derived from neural signal components in the brain. The middle stage (within the blue shaded box) demonstrates the model’s dynamic internal state. c*^ext^* represents the combination of the external inputs, c*^int^* represents the internal connections within the model, *u* denotes the model’s internal state, and *a* signifies the model’s output activity. The final stage shows the output activity. **(C)** This part provides a graphical representation of the three levels of controlled variability for the Onset Delay, the RoR, and the MaxVal across different inputs (refer to Table 1).

We used the gap task to align with the Coe et al. (2019) model, which is based on this paradigm. The gap saccadic task simply asks the subject to make a saccade toward a 1-degree target that appears after the end of the fixation period and gap period (Figure 1A). We used a 200 ms fixation period, 200 ms gap period and 6 degrees for visual stimulus eccentricity. The target location was randomly chosen from eight equidistant positions, spaced at 45-degree intervals along a radial visual angle, for each trial. A 2-degree-radius invisible eye window was centered on the fixation and the target stimuli. During fixation, the gaze had to remain within the fixation window, and the saccade needed to land within the target window. A black dot with a white center was used as the stimulus. Successful responses are rewarded with a presentation of a marmoset photo and a 0.05 ml reward; prepared by mixing baby supplement banana pudding (Kewpie Corp., Japan) with banana-flavored Mei-balance (Meiji Holdings Co., Japan). If the response was incorrect, a one-frame red screen was flashed.

To further align with the Coe et al. (2019) model, we specifically isolated and analyzed only horizontal saccades. We collected 1112, 8318 and 436 trials from marmoset P, J and M respectively, and 94, 96 and 82 trials from human W, C and H respectively.

### Eye tracking, calibration, saccade detection and saccade reaction time (SRT) quantification

We tracked the eyes of marmosets and humans binocularly using an Eyelink 1000 Plus and Eyelink 1000 (SR Research, Canada), respectively, at a sampling frequency of 500 Hz. Both pupil and corneal reflection information were utilized to ensure better accuracy. Eye position calibration and validation followed the method described by Chen et al. (2021). For saccade detection, we used U’n’Eye (Bellet et al., 2019). After manually labeling around 200 trials, the data were used to train and validate U’n’Eye for optimal saccade detection. Rather than relying solely on peak velocity or a predefined threshold, U’n’Eye extracts multiple features, learning typical relationships between amplitude and duration. It detects saccade start and end based on a holistic analysis of eye movement, rather than a single velocity peak. Once trained, the network labeled the remaining pre-processed eye data. The SRT of the extracted saccade is defined as the time between target onset and saccade initiation.

### The neural field model and the component-based inputs

We used (Coe et al., 2019) model which employs multiple component-based inputs to simulate the potential neural processes underlying saccade generation in marmosets. Briefly, the feature space emulating the intermediate layer of the superior colliculus (SC) was represented by a 1D-field with a vector of 100 nodes (*N* = 100), spanning from −5 mm (far left) to 5 mm (far right), with zero indicating the center on the SC surface, to simulate horizontal eye movements. As shown in Figure 1B, each of the 8 inputs [externally triggered: Visual Transient (①), Automated Motor (②), and Automated Fixation (③); internally driven: Voluntary Motor (④), Voluntary Fixation (⑤), Voluntary Preparation (⑥), Inhibitory Gate (⑦), and Peripheral Inhibition (⑧)] was characterized by four parameters: onset delay, rate of response (RoR), maximum strength value (MaxVal), and center of activity (μ) (Figure 1C), and updated every millisecond (Equations 1 and 2).


(1) inputi(t+△t)=inputi(t)+(k*iμRoR*△t)

(2) $$k_{i}^{\mu }=a\,m\,p*e\,x\,p\,{\frac{-\left(\text{min}\,\left(\left|i\,△\,x-\mu \right|,N-\left|i\,△\,x-\mu \right|\right)\right)}{2\,\gamma^{2}}}^{2}$$

where node index (*i*) indicates position in space (1:100), △t indicated the increment of time (△*t* = 1 ms for all simulations). The value of κ at each node was determined by the node’s distance from the center of activity (γ), using a Gaussian profile with a standard deviation (γ) of 0.6 and an amplitude (amp) of 1.05. The numerator in the exponential represents the minimum distance of a node from the center of the Gaussian on a toroidal feature space, avoiding boundary conditions—a common technique in neural field computations.

### The model inputs

The model incorporates eight inputs that emulate neural activity patterns observed in the primate brain during saccade tasks, as detailed by Coe et al. (2019). Briefly, Visual Transient Input **①** mimics activity in the magnocellular LGN and superficial layers of the SC, producing a brief burst shortly after the visual stimulus onset. Automated Motor Input **②** represents an externally triggered, self-propagating motor command for saccades to peripheral stimuli, with timing based on lateral intraparietal area recordings from macaques. Automated Fixation Input **③** also reflects an externally triggered component but pertains to currently foveated stimuli, modeled after parietal cortex activity. Voluntary Motor Input ④ is internally triggered without stimulus appearance, modeled after frontal cortex activity. Voluntary Fixation Input **⑤** initiates commands to maintain fixation regardless of stimulus presence, based on frontal cortex fixation activity. Voluntary Preparation Input **⑥** is modeled after activity in the supplementary and frontal eye fields. Inhibitory Gate Input **⑦** creates a field-wide inhibition barrier to suppress motor commands, which opens selectively to release commands by removing inhibition in specific visual field areas, similar to the substantia nigra pars reticulata (SNr) mechanisms. Finally, Peripheral Inhibition Input **⑧** provides inhibition in the periphery to support fixation, lifting this inhibition when a motor command is prepared to allow excitatory signals to propagate through the SCi. This input is modeled after omni-directional pause neurons and universal pausers in the basal ganglia.

### Dynamic integration of saccadic activity

The model composed of three stages; the input, the integration and the output stages, Figure 1B. The output activity ***a*** was calculated for each time point (*t*) as a nonlinear function of its internal state (***u***) using a sigmoidal function:


(3) $$\text{a}\,\left(t\right)=\frac{1}{1+\text{exp}\,\left(-\beta \,u\,\left(t\right)\right)}$$

The steepness of the sigmoid was set to β = 0.09. Saccade initiation threshold states when the output activity at any non-central location reached 0.7.


(4) $${\text{u}}_{i}\,\left(t+△\,t\right)=\left(1-\frac{△\,t}{\tau }\right)*{\text{u}}_{i}\,\left(t\right)+\frac{△\,t}{\tau }*\left(c_{i}^{e\,x\,t}\,\left(t\right)+c_{i}^{i\,n\,t}\,\left(t\right)\right)$$

where △t = 1 ms is the time discretization step, *i* = 1:100 denotes the spatial index, Tau (τ) = 4 is the time scale constant, the external contribution (c*^ext^*) is a vector representing the sum of the eight external inputs to the model, and the internal contribution (c*^int^*) is a vector representing the connections across the model (Equation 5).


(5) $$c^{i\,n\,t}\,\left(t\right)=W*\text{a}\,\left(t\right)$$

(6) W=(G-m)*△⁢x

(7) $$G_{i,j}=s\,f*\text{exp}\,\left(\frac{-{\left(\min\left(\left|i-j\right|,N-\left|i-j\right|\right)*△\,x\right)}^{2}}{2\,\sigma^{2}}\right)$$

(***a***) is the model’s current activity and W is a laterally-inhibitory weight matrix. The matrix W defined in Equation 6, is positive for nearby nodes and negative for distant ones. It is derived from a Gaussian matrix G (Equation 7) which is shifted by 80% of its maximum value [*m* = 0.8 × max(G)] and scaled by △x. Here, △x = 10/N represents the distance between nodes, with the feature space ranging from −5 to 5 mm of SC tissue. The Gaussian width is set to σ = 0.85 with a scaling factor sf = 74.7. Both the c*^int^* and ***u*** vectors were reset to −30 to represent a negative membrane potential, ensuring that each trial was unaffected by the previous one.

### Optimizing model parameters

We followed Coe et al. (2019) and implemented a jointly varying onset delay for the internally driven inputs. We kept the RoR for the Voluntary Preparation input dependent on its MaxVal and equal for both locations (μ). We also kept the MaxVal of the voluntary motor input to continue to rise and compete until a saccade was made. We incorporated neurophysiological findings and behavioral evidence from marmosets to modify the remaining input parameters (Cheong and Johannes Pietersen, 2014; Feizpour et al., 2021), informed by comparable data from macaque neural recordings (Schmolesky et al., 1998; McAlonan et al., 2008). Despite limited specific research on marmosets, this approach allowed us to adjust parameters based on insights drawn from related macaque studies. The modifications we made are summarized in Table 1. The justifications for the changes we employed are described in the sections “Results and discussion.”

**TABLE 1:** The possible settings used for each input for each trial. The changes made at each step are color-coded as described in the text and illustrated in Figure 3.

	Onset delay (ms)	RoR (%)	MaxVal
**Marmosets 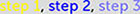 **			
1. Visual transient	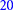	10, 15, 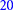	8
2. Automated motor	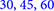	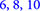	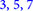
3. Automated fixation	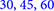	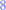	6
4. Voluntary motor	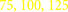	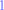 , 10, 20	Dependent
5. Voluntary fixation	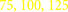	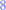	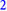 , 4, 6, 8
6. Voluntary preparation	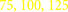	Dependent	4, 6, 8
7. Inhibitory gate	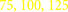	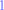 , 10, 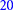	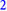 , 4, 6, 8
8. Peripheral inhibition	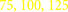	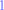 , 10, 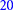	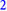 , 4, 6, 8
	**Onset delay (ms)**	**RoR (%)**	**MaxVal**
**Humans 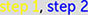 **			
1. Visual transient	50	10, 15, 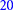	8
2. Automated motor	60, 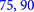	4, 6, 8	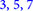
3. Automated fixation	60, 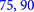	10	6
4. Voluntary motor	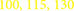	5, 10, 15	Dependent
5. Voluntary fixation	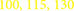	10	4, 6, 8
6. Voluntary preparation	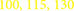	Dependent	4, 6, 8
7. Inhibitory gate	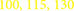	5, 10, 15	4, 6, 8
8. Peripheral inhibition	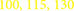	5, 10, 15	4, 6, 8

Like Coe et al. (2019) we also introduced multiple levels of controlled variability by assigning various values to each parameter (onset delay, Rate of Response (RoR), and MaxVal) tailored for marmosets. As indicated in Table 1, there were 3 values for 10 attributes and 4 values for 3 attribute, resulted in (3^10^ × 4^3^) = 3,779,136 possible combinations for marmosets. For humans, there were 3 values for 13 attributes resulted in 3^13^ = 1,594,323 possible combinations for humans, each representing an individual trial.

## Results

### Identification of anticipatory, express and regular saccades

**FIGURE 2:**
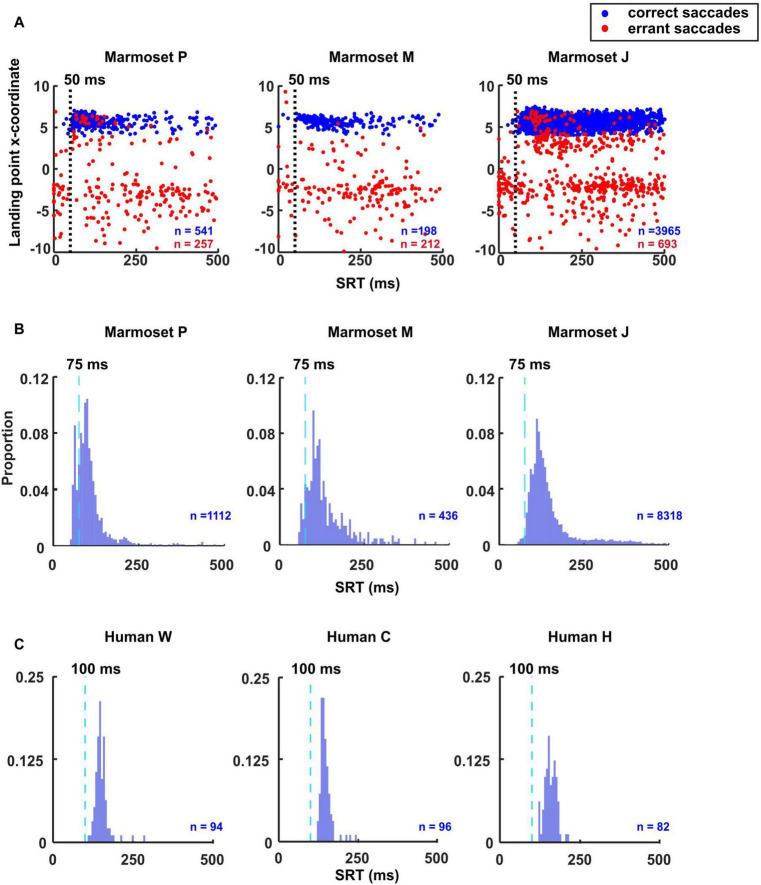
Identification of anticipatory, express and regular saccade thresholds. **(A)** Scatterplots demonstrate the method used to categorize anticipatory saccades in marmosets. Each plot represents saccades made toward the rightward target. The landing point x-coordinate is plotted against primary saccade latency measured from the onset of the visual target at 6 deg eccentricity. Correct saccades are presented in blue and errant saccades are in red. The vertical line in each plot represents the latency boundary, determined by the point where the number of errant saccades exceeds the number of correct ones. Short latencies to the left of the boundary represent anticipatory saccades that were not visually driven, while latencies to the right represent visually driven express and regular saccades. SRT histograms plotted with 6 ms bins. The dashed lines indicate the threshold between express and regular saccade in marmosets **(B)** and humans **(C)**.

Anticipatory saccades are eye movements made before a visual target appears, reflecting the expected location of the target. They are a type of voluntary saccade and typically have shorter SRTs than visually guided saccades. To help determine anticipatory saccades threshold, we followed a similar approach as (Kalesnykas and Hallett, 1987) and plotted the reaction time vs the landing point x-coordinate for the successful and errant saccades (Figure 2A) to estimate the threshold between the visually driven (express and regular) vs non-visually driven (anticipatory) saccades.

On the other hand, saccades obtained during a gap task with a 200 ms gap often feature a bimodal distribution of SRT. One is attributed to express saccades and the other to regular saccades (Fischer and Boch, 1983; Fischer and Ramsperger, 1984). The exact timing of express latency saccades varies across subjects but is generally considered to be 80–130 ms for humans (Saslow, 1967; Fischer and Ramsperger, 1984; Kingstone and Klein, 1993; Biscaldi et al., 1996; Chen et al., 2021) and 70–120 ms for macaques (Paré and Munoz, 2001). Saccades longer than this are referred to as regular saccades. Previous studies did not precisely define the range in marmosets but showed that their SRT is shorter than humans (Ma et al., 2020; Chen et al., 2021). To estimate the border between express and regular saccades, we plotted the SRT histogram to help determine the minimum latency for regular saccades (Figure 2B).

In contrast, human subjects in this study exhibited almost no errant or express saccades, Supplementary Figure 1. Nonetheless, we plotted the SRT histogram to determine the minimum latency for regular saccades (Figure 2C). Based on the results depicted in Figure 2, we categorized express saccades as those occurring in times shorter than 75 ms and longer than 50 ms. Therefore, we determined the minimum threshold for regular saccades to be 75 ms for marmosets and 100 ms for humans.

### Marmosets have shorter saccade reaction time (SRT) than humans

**FIGURE 3:**
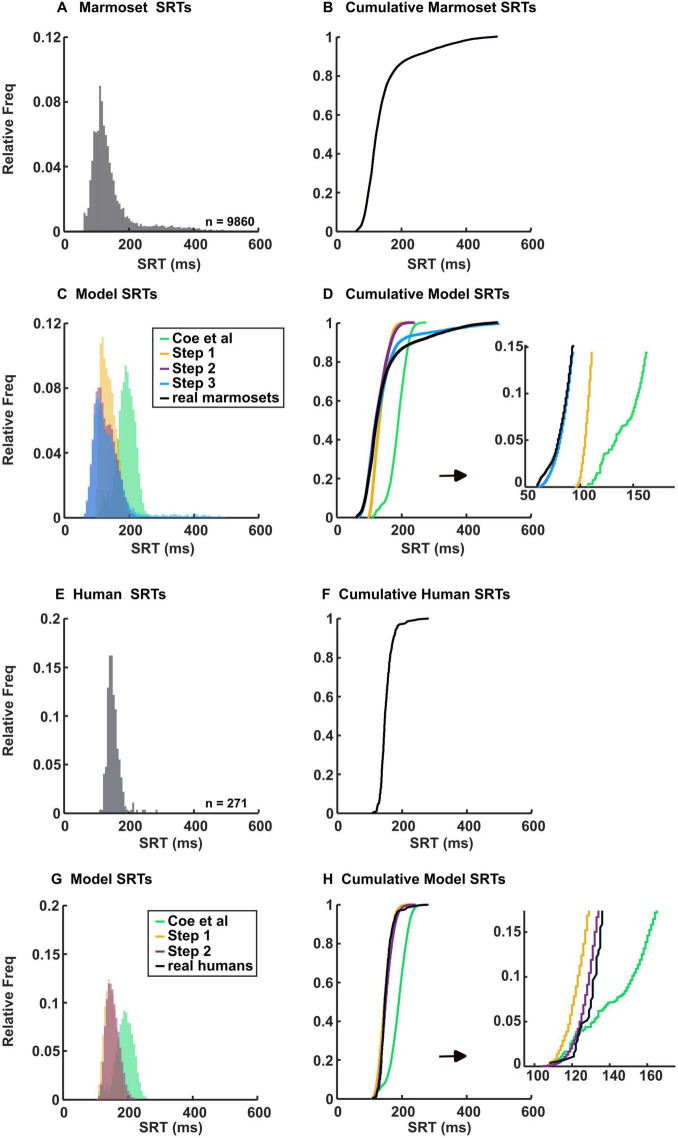
Histograms of marmoset and human SRTs and their model simulations, plotted with 6 ms bins. Actual **(A)** and cumulative **(B)** histograms show the percentages of saccades and their latencies from the 3 marmosets performing the gap task. Simulated **(C)** and cumulative **(D)** histograms showing percentages of saccades and their latencies from the model. Step 1 data consists of modifications to the onset delay of inputs 4–8. Step 2 data includes modifications from step 1 plus adjustments made to alter the visual response. Step 3 data incorporates modifications from steps 1 and 2, along with changes made to adjust the variability in SRT. Actual **(E)** and cumulative **(F)** histograms showing the percentages of saccades and their latencies from the 3 humans performing the gap task. Simulated **(G)** and cumulative **(H)** histograms showing percentages of saccades and their latencies from the model. Step 1 data consists of modifications to the onset delay of inputs 4–8. Step 2 data includes modifications from step 1, along with adjustments made to inputs 1 and 2 to maintain consistency with our approach for marmosets. Each simulated set of data is composed of 20,000 random trials.

We combined data from the same species to create species-specific fitted parameters. The SRTs of saccades collected from marmosets (Figures 3A, B) and humans (Figures 3E, F) are presented in the histograms and cumulative distribution function (CDF) of SRTs. By examining the SRT distribution of marmosets and humans (Figure 3), it becomes immediately apparent that marmosets have shorter SRTs (*p* = 1.4 × 10^–31^, Wilcoxon rank-sum test), as indicated by the earlier part of the CDF. The median of SRT is 122 ms in marmosets and 147 ms in humans. The shortest SRT is 61 ms for marmosets and 108 ms for humans.

To start the simulation, we initially considered the onset delay of inputs 4–8 (Table 1), which represents the cut-off between express and regular saccade, as the most apparent parameter to change. In Coe et al. (2019) they used a regular saccade cut-off of 140 ms, (Figure 3D, green line). However, as described earlier, the marmoset SRT is shorter, necessitating a lower cut-off. Thus, we adjusted this parameter to 75, 100 and 125 ms for marmoset (step 1) while using the same parameters outlined by Coe et al. (2019) and ran the simulation. Despite using a shorter onset delay, the model did not accurately capture the shape of the marmoset SRT distribution, particularly the short SRTs and the long tail (Figures 3C, D, yellow line). The R^2^ value was 0.89 and the mean squared error (MSE) was 0. 009. This discrepancy underscores the distinct neural processes involved in saccade generation in marmosets and prompted us to explore the reasons behind their shorter yet variable SRTs.

Because our human subjects also had a shorter SRT than those in Coe et al. (2019), we lowered the cut-off for regular saccades by shortening the onset delay to 100, 115 and 130 ms (step 1, Figures 3G, H, yellow line). The R^2^ value was 0.97 and the MSE was 0.0034. We included data from our own human subjects to test the reproducibility of the Coe et al. (2019) model and to demonstrate that our modifications are specific to marmosets, given their distinct SRT behavior compared to humans. Notably, we used U’n’Eye for saccade detection instead of older methods relying on velocity thresholds. U’n’Eye may be more sensitive to subtle eye movements that do not reach the strict thresholds of traditional methods, allowing it to capture saccades initiated before significant velocity increases. This could explain the shorter SRTs observed in our human subjects compared to those reported by Coe et al. (2019); however, marmosets still showed shorter SRTs than humans using the same detection method.

Shorter SRT implies that the perception and the processing of visual stimuli are fast in marmosets. Indeed, previous studies have shown that the response latency in V1 is shorter in marmosets than in macaques (Schmolesky et al., 1998; Feizpour et al., 2021). Furthermore, recording experiments on the LGN suggested that magnocellular cells in marmosets respond more quickly to visual stimuli compared to those in macaques (Schmolesky et al., 1998; McAlonan et al., 2008; Cheong and Johannes Pietersen, 2014). Building on this, we adjusted the onset delay for the visual transient input to 20 ms for marmosets, while maintaining it at 50 ms for humans.

Due to the reduction in onset delay for the visual transient, we correspondingly adjusted the automated motor input to have a minimum of 30 ms for marmosets. Besides, recordings in the LIP of marmosets have shown that neurons fire at least as quickly, if not faster, than in macaques, which was used by Coe et al. (2019) to model this input (Barash et al., 1991; Selvanayagam et al., 2024). Thus, we set the maximum onset delay to 60 ms. Therefore, while marmosets demonstrate quicker processing in early visual stages, the complexity of the saccadic system and the characteristics of downstream areas (ex. LIP) can influence overall SRTs, contributing to the observed variability. For humans, we retained the 60 ms delay as specified in Coe et al. (2019), while introducing a 15 ms variation to range between 60, 75, and 90 ms.

Another contributing factor to shorter SRTs could be weaker inhibition leading to quicker disinhibition. For instance, marmosets appear to face greater challenges than macaques when attempting to complete the standard antisaccade task. This difficulty likely stems from their reduced ability to inhibit reflexive eye movements (Johnston et al., 2019). Consequently, we augmented the disinhibition by adding 2 to the MaxVal of inhibitory gate and peripheral inhibition inputs.

Moreover, it is known that express saccades frequently occur when active fixation or directed visual attention is disengaged 200 ms before the saccade target appears, and almost completely abolished if fixation or attention is still engaged when the saccade target appears (Mayfrank et al., 1986; Fischer and Weber, 1993). This suggests that fast saccades occur when visual attention has already been disengaged from its previous locus before the saccade target onset. Another study demonstrated that subjects with a high propensity for making express saccades were impaired at suppressing visually-evoked saccades during fixation periods or when preparing voluntary saccades to other locations (Biscaldi et al., 1996). This implies a deficit in the ability to maintain fixation and inhibit reflexive saccades triggered by abrupt visual onsets, therefore, we lessened fixation strength by adding 2 to the MaxVal of the voluntary fixation to simulate cases of weaker fixation.

Additionally, some previous research suggested that marmosets have a smaller pool of neurons than macaques (or humans). For instance, (Pandey et al., 2020) showed that marmosets can make rapid, reflexive head turns in response to natural stimuli, with peak velocities exceeding 1,000°/s. This rapid, saccadic head-orienting behavior suggests that they have a fast build-up of neuronal activity to support such quick and precise movements. Furthermore, (Chaplin et al., 2017) have shown that marmosets’ MT is 4–5 times smaller than in macaques and that their cerebral cortex contains few sulci, with most visual areas like MT fully exposed on the outer cortical surface. Despite the difference in scale, they possess similar structures and areas found in macaques and humans (Walker et al., 2017). Thus, we increased the RoR to a maximum of 20% for input 1, to 6%, 8%, and 10% for input 2, and to a maximum of 20% for inputs 7 and 8, assuming faster build-up activity.

These adjustments collectively (step 2) brought the simulation results closer to replicating the short SRTs observed in marmosets (Figures 3C, D, purple line). The R^2^ value was 0.94 and the MSE was 0.005.

We used the same RoR values for input 1 for humans as we did for marmosets, as well as the same MaxVal for input 2 (step 2, Table 1), (Figures 3G, H, purple line). The R^2^ value was 0.98 and the MSE was 0.0029.

### Marmosets exhibit greater variability in SRT resulting in more delayed saccades

As Figures 3A, B illustrate, marmosets exhibit greater variability in SRT compared to humans (Figures 3E, F), resulting in a longer, extended tail of the CDF and more saccades having SRT > 250 ms (9.4% vs 0.4%, *p* = 3.7 × 10^–7^, Wilcoxon rank-sum test). With the parameters outlined in Coe et al. (2019) the model initially failed to replicate the later SRT distribution observed in marmosets.

In exploring potential causes for slower SRT, one study inferred that the central visual field is better represented or has stronger neural representations compared to the peripheral visual field in the marmoset brain, consistent with their small eye size (Yu et al., 2015). In parallel to that, research has stated that glaucomatous eyes exhibited significantly lengthened SRTs compared to healthy controls and the authors suggest that peripheral sensitivity loss in glaucoma may impair the ability to disengage fixation and initiate saccades, leading to slower SRTs (Thepass et al., 2021).

Based on that, we hypothesized that disengagement from fixation might not always occur swiftly. To address this, we introduced a minimum RoR of 1% for the inhibitory gate and peripheral inhibition inputs and enhanced the RoR of automated fixation and voluntary fixation inputs to be 8%.

Other potential causes include a slower building-up of the voluntary motor signal or a more variable onset delay for the build-up activity. Thus, we introduced a minimum RoR of 1% for the voluntary motor input.

These additional adjustments (step 3), combined with our previous modifications, brought the simulation results closer to replicating the observed SRTs in marmosets (Figure 3D, blue line). The R^2^ value was 0.99 and the MSE was 0.0006.

Finally, to demonstrate that the modifications in step 3 improved the model’s ability to capture SRT distribution in marmosets compared to step 1 (which achieved an R^2^ of 0.89), we tested the Wasserstein (Earth Mover’s) Distance, sensitive to overall shape differences in the distributions. The Normalized Wasserstein Distance for step 3 was 694,254.5, compared to 1,255,615.5 for step 1, representing a twofold improvement in performance. Furthermore, we compared the quantiles of the two distributions to assess the model’s fit and identify deviations from expected distributions. As shown in Supplementary Figure 2A, the modifications in step 3 brought the model closer to matching the real marmoset data. We also compared the CDFs of the real marmoset data with the simulated models (step 3 vs. step 1). As depicted in Supplementary Figure 2B, the step 3 model more accurately replicates the real marmoset SRT distribution compared to step 1.

To further validate our model’s performance in capturing individual variations, we analyzed the SRT data for each marmoset and assessed how well the model aligned with the real data. As shown in Supplementary Figure 3, the model captures the shape of each marmoset’s SRT distribution well, with consistently good R^2^ and MSE values.

With the refined parameters listed in Table 1, we successfully replicated both the shorter and more variable SRT observed in marmosets.

## Discussion

Our marmoset-fitted parameters offer insights into the factors influencing differences in the temporal processing of afferent signals across different brain areas. These differences contribute to distinct behavioral outcomes in marmosets compared to humans. This understanding can help clarify why it’s easier to train marmosets on certain tasks but not the other and why they show varying levels of success in task performance compared to other species.

Below, we elaborate on the rationale behind our observations and the modifications to the parameters made in this study.

### Marmosets have an earlier visual response

Various studies on marmosets performing visually guided saccadic task have consistently shown shorter SRT compared to macaques or humans (Ma et al., 2020; Chen et al., 2021; Amly et al., 2023). Our findings corroborate these results, clearly demonstrating that marmosets exhibit shorter SRTs than humans (Figures 2, 3).

One possible reason is to have earlier perception and the processing of visual stimuli, as elaborated in the results section earlier. Alternatively, one would also think that a smaller brain may react faster than a bigger brain. It is generally considered that smaller animals generally have greater temporal resolution of vision, meaning they can detect flickering light at higher frequencies than larger animals. This is linked to higher metabolic rates in smaller animals (Tomasik, 2016). In general, birds, appear to have faster reaction times. This is attributed to their high metabolic rates and the fact that smaller organisms tend to perceive changes on shorter timescales (Potier, 2023).

Furthermore, one study suggested that having a larger number of cortical neurons, which is associated with larger brain size, can lead to longer neural processing times. The study found that the enlargement of human brain size, or more accurately, the increase in the number of cortical neurons developing throughout primate evolution, correlated with an increase in the dwell time of auditory cortical processing in humans (Itoh et al., 2022). This finding suggests that the evolutionary prolongation of cortical processing time might have occurred not only in audition but also in other sensory modalities, including vision.

With a larger neuronal pool, the organization and efficiency of information processing can impact the speed of action. In some cases, it might exhibit less efficient processing due to increased noise or competition among neurons for resources, which could also contribute to delays in action. Narayanan and Laubach (2009) proposed a model where two competing pools of neurons in the frontal cortex and motor cortex represent the “prepotent response” to execute an action and the “proactive inhibition” to delay that action. A larger pool of neurons accumulating activity for the proactive inhibition could lead to a longer delay before the action is triggered. He also suggested that dynamic changes and interactions within large neuronal population activity, rather than just single neuron activity, may be critical for temporal preparation and delaying actions appropriately based on task rules. Furthermore, (Senn et al., 2023) introduced a neuronal “least-action” principle where cortical pyramidal neurons prospectively minimize errors, implying their voltage dynamics effectively look ahead in time. A larger recurrent network of such neurons could implement more complex prospective coding leading to delayed motor outputs. Furthermore, (Izhikevich, 2006; Egger et al., 2020) discussed that local axonal conduction delays between neurons in a feedforward polychronous network can enforce precise timing of neural activity sequences. A larger network with more interconnected neurons will accumulate more conduction delays, resulting in a longer delay for the overall activity sequence.

Thus, several theories propose that increasing the size of interconnected neuron pools, incorporating more synapses, conduction delays, recurrent interactions, and prospective coding mechanisms, can result in a longer delay from sensory input to motor output in neural circuits involved in action preparation and execution. A larger neuron population scale appears to enable greater temporal integration and delay of actions.

Overall, these findings suggest a trade-off between the advantages of larger brain size, such as enhanced cognitive capabilities and finer visual processing, and the potential drawback of slower processing speeds for certain sensory inputs, including visual information, which could explain the shorter SRT observed in marmosets.

### Marmosets might have a variable level of inhibition

Another possible factor that might contribute to the shortening of SRT is having weaker inhibition. One vital brain area to think of is the basal ganglia (BG), which plays a role in suppressing the automatic triggering of express saccades. Thus, BG dysfunction or a decrease in its functionality can increase the incidence of faster saccades by reducing the normal suppression of these automatic saccades.

Previous results suggested the external globus pallidus (GPe), through the indirect pathway, can exert an inhibitory gating influence over saccade-related activity in the SNr. This inhibitory gating may contribute to the regulation of saccade initiation and suppression of unwanted saccades by the BG oculomotor circuit (Kato and Hikosaka, 1995). In addition, the BG, via the SNr output, facilitates or gates the initiation of desired saccades while suppressing others (Handel and Glimcher, 1999). Another study found that when the strength of the GPe to striatum connection is increased, the stop-signal reaction time decreases (Wei and Wang, 2016). This suggests that stronger inhibition from the GPe to the striatum leads to faster stopping times, which implies that weaker inhibition could lead to shorter reaction times for go responses. Furthermore, (Schmidt et al., 2013) discussed the role of BG pathways in canceling actions, which is related to response inhibition and timing. It mentions “weak” shunting inhibition, suggesting that the strength of inhibition plays a role in action control.

On the other hand, some studies showed that in Parkinson’s disease (PD) patients, the gap paradigm led to the generation of express-like saccades, similar to express saccades seen in normal subjects and the percentage of express-like saccades in the gap condition was significantly higher compared to age-matched control subjects (Roll et al., 1996). This implies that the dysfunction of the BG in PD may reduce the suppression of the automatic triggering of express saccades, allowing the gap paradigm to more readily elicit express-like saccades. Another study showed that PD patients have an impaired ability to exert voluntary control over oculomotor reflexes like the visual grasp reflex (van Koningsbruggen et al., 2009; Srivastava et al., 2014). The results indicate that the BG play an important role in implementing excitatory and inhibitory control over primitive oculomotor reflexes. Any dysfunction may compromise this voluntary control, leading to an inability to adequately suppress reflexive saccades.

Thus, the collective evidence from these results suggests that the strength of inhibitory signals in the BG can influence response times. Weaker inhibition generally seems to be associated with shorter response times or a higher likelihood of making rapid responses.

Based on that, we propose that marmosets might have weaker inhibition strength, which allows them to generate shorter SRT. This might also explain why it is hard to train marmosets on antisaccade tasks or delayed saccade tasks with long delay periods (Johnston et al., 2019; Amly et al., 2023). Having a weaker level of inhibition makes them more susceptible to breaking fixation and/or incapable of suppressing unwanted saccades.

However, despite the generally weaker level of inhibition in marmosets, it appears that the inhibition level is not consistently weak but rather variable. This variability contributes to the long tail observed in the CDF (Figures 3A, B). Research shows that the BG exert inhibitory control over saccade initiation, which the SC must overcome. Increased inhibitory output from the BG, particularly the SNr, can delay saccade initiation by maintaining inhibition on the SC (Hikosaka et al., 2000). Krishnan et al. (2011) demonstrated through a computational model that longer SRTs occur when the BG fail to effectively reduce inhibition, aligning with observations in PD patients. This leads to increased inhibition compared to a normal state where inhibition can be flexibly adjusted.

## Data Availability

The raw data supporting the conclusions of this article will be made available by the authors, without undue reservation.

## Supplementary material

Supplementary Figure 1 Identification of anticipatory saccade thresholds in humans. Each scatterplot represents saccades made toward the rightward target. The landing point x-coordinate is plotted against primary saccade latency measured from the onset of the visual target at 6 deg eccentricity. Correct saccades are presented in blue and errant saccades are in red. No early errant saccades were made by human subjects.

Supplementary Figure 2 Statistical figures demonstrating the superiority of step 3 modifications over step 1. **(A)** The Q-Q plot compares the quantiles of the simulated data to the real marmoset SRTs, assessing the fit and identifying deviations. In step 1, points in the Q-Q plot deviate from the straight line, indicating differences between the distributions. In contrast, the points in step 3 fall approximately along a straight line, suggesting that the distributions are more closely aligned. **(B)** Differences in the cumulative distribution functions between the real marmoset data and the simulated models were also evaluated. A smaller difference in CDFs indicates a better fit, with the step 3 model more effectively replicating the real marmoset SRT distribution than step 1.

Supplementary Figure 3 Cumulative distribution of individual Marmoset SRTs and our Model. The figure illustrates how closely the model captures the overall SRT distribution of individual marmosets.

## References

[B1] Amly W. Chen C. Y. Onoe H. Isa T. (2023). Dissecting errors made in response to externally and internally driven visual tasks in the common marmosets and humans. *bioRxiv* [Preprint]. 10.1101/2021.08.29.458139

[B2] Barash S. Bracewell R. M. Fogassi L. Gnadt J. W. Andersen R. A. (1991). Saccade-related activity in the lateral intraparietal area. I. Temporal properties; comparison with area 7a. *J. Neurophysiol.* 66 1095–1108. 10.1152/jn.1991.66.3.1095 1753276

[B3] Bellet M. E. Bellet J. Nienborg H. Hafed Z. M. Berens P. (2019). Human-level saccade detection performance using deep neural networks. *J. Neurophysiol.* 121 646–661. 10.1152/jn.00601.2018 30565968

[B4] Biscaldi M. Fischer B. Stuhr V. (1996). Human express saccade makers are impaired at suppressing visually evoked saccades. *J. Neurophysiol.* 76 199–214. 10.1152/jn.1996.76.1.199 8836219

[B5] Chaplin T. A. Allitt B. J. Hagan M. A. Price N. S. C. Rajan R. Rosa M. G. P. (2017). Sensitivity of neurons in the middle temporal area of marmoset monkeys to random dot motion. *J. Neurophysiol.* 118 1567–1580. 10.1152/jn.00065.2017 28637812 PMC5596136

[B6] Chen C.-Y. Matrov D. Veale R. Onoe H. Yoshida M. Miura K. (2021). Properties of visually guided saccadic behavior and bottom-up attention in marmoset, macaque, and human. *J. Neurophysiol.* 125 437–457. 10.1152/jn.00312.2020 33356912

[B7] Cheong S. K. Johannes Pietersen A. N. (2014). Antidromic latency of magnocellular, parvocellular, and koniocellular (Blue-ON) geniculocortical relay cells in marmosets. *Vis. Neurosci.* 31 263–273. 10.1017/S0952523814000066 24703370

[B8] Clarke H. F. Hill G. J. Robbins T. W. Roberts A. C. (2011). Dopamine, but not serotonin, regulates reversal learning in the marmoset caudate nucleus. *J. Neurosci.* 31 4290–4297. 10.1523/JNEUROSCI.5066-10.2011 21411670 PMC3083841

[B9] Coe B. C. Trappenberg T. Munoz D. P. (2019). modeling saccadic action selection: Cortical and basal ganglia signals coalesce in the superior colliculus. *Front. Syst. Neurosci.* 13:3. 10.3389/fnsys.2019.00003 30814938 PMC6381059

[B10] Egger R. Tupikov Y. Elmaleh M. Katlowitz K. A. Benezra S. E. Picardo M. A. (2020). Local axonal conduction shapes the spatiotemporal properties of neural sequences. *Cell* 183:537–548.e12. 10.1016/j.cell.2020.09.019 33064989 PMC7577554

[B11] Feizpour A. Majka P. Chaplin T. A. Rowley D. Yu H.-H. Zavitz E. (2021). Visual responses in the dorsolateral frontal cortex of marmoset monkeys. *J. Neurophysiol.* 125 296–304. 10.1152/jn.00581.2020 33326337

[B12] Fischer B. Boch R. (1983). Saccadic eye movements after extremely short reaction times in the monkey. *Brain Res.* 260 21–26. 10.1016/0006-8993(83)90760-6 6402272

[B13] Fischer B. Ramsperger E. (1984). Human express saccades: Extremely short reaction times of goal directed eye movements. *Exp. Brain Res.* 57:1145. 10.1007/BF00231145 6519226

[B14] Fischer B. Weber H. (1993). Express saccades and visual attention. *Behav. Brain Sci.* 16 553–567. 10.1017/S0140525X00031575

[B15] Handel A. Glimcher P. W. (1999). Quantitative analysis of *Substantia nigra* pars reticulata activity during a visually guided saccade task. *J. Neurophysiol.* 82 3458–3475. 10.1152/jn.1999.82.6.3458 10601475

[B16] Hikosaka O. Takikawa Y. Kawagoe R. (2000). Role of the basal ganglia in the control of purposive saccadic eye movements. *Physiol. Rev.* 80 953–978. 10.1152/physrev.2000.80.3.953 10893428

[B17] Itoh K. Konoike N. Nejime M. Iwaoki H. Igarashi H. Hirata S. (2022). Cerebral cortical processing time is elongated in human brain evolution. *Sci. Rep.* 12:1103. 10.1038/s41598-022-05053-w 35058509 PMC8776799

[B18] Izhikevich E. M. (2006). Polychronization: Computation with spikes. *Neural Comput.* 18 245–282. 10.1162/089976606775093882 16378515

[B19] Jendritza P. Klein F. J. Rohenkohl G. Fries P. (2021). Visual neuroscience methods for marmosets: Efficient receptive field mapping and head-free eye tracking. *Eneuro* 8:ENEURO.0489-20.2021. 10.1523/ENEURO.0489-20.2021 PMC8143020 33863782

[B20] Johnston K. D. Barker K. Schaeffer L. Schaeffer D. Everling S. (2018). Methods for chair restraint and training of the common marmoset on oculomotor tasks. *J. Neurophysiol.* 119 1636–1646. 10.1152/jn.00866.2017 29364068

[B21] Johnston K. Ma L. Schaeffer L. Everling S. (2019). Alpha oscillations modulate preparatory activity in marmoset area 8ad. *J. Neurosci.* 39 1855–1866. 10.1523/JNEUROSCI.2703-18.2019 30651331 PMC6407288

[B22] Kalesnykas R. P. Hallett P. E. (1987). The differentiation of visually guided and anticipatory saccades in gap and overlap paradigms. *Exp. Brain Res.* 68:5238. 10.1007/BF00255238 3691690

[B23] Kato M. Hikosaka O. (1995). Function of the indirect pathway in the basal ganglia oculomotor system: Visuo-oculomotor activities of external pallidum neurons. *Front. Neurol. Neurosci.* 14:423603. 10.1159/000423603

[B24] Kingstone A. Klein R. M. (1993). What are human express saccades? *Percept. Psychophys.* 54 260–273. 10.3758/BF03211762 8361841

[B25] Krishnan R. Ratnadurai S. Subramanian D. Chakravarthy V. S. Rengaswamy M. (2011). Modeling the role of basal ganglia in saccade generation: Is the indirect pathway the explorer? *Neural Netw.* 24 801–813. 10.1016/j.neunet.2011.06.002 21726978

[B26] Ma L. Selvanayagam J. Ghahremani M. Hayrynen L. K. Johnston K. D. Everling S. (2020). Single-unit activity in marmoset posterior parietal cortex in a gap saccade task. *J. Neurophysiol.* 123 896–911. 10.1152/jn.00614.2019 31967927

[B27] Mayfrank L. Mobashery M. Kimmig H. Fischer B. (1986). The role of fixation and visual attention in the occurrence of express saccades in man. *Eur. Arch. Psychiatry Neurol. Sci.* 235 269–275. 10.1007/BF00515913 3732337

[B28] McAlonan K. Cavanaugh J. Wurtz R. H. (2008). Guarding the gateway to cortex with attention in visual thalamus. *Nature* 456 391–394. 10.1038/nature07382 18849967 PMC2713033

[B29] Miller C. T. Freiwald W. A. Leopold D. A. Mitchell J. F. Silva A. C. Wang X. (2016). Marmosets: A neuroscientific model of human social behavior. *Neuron* 90 219–233. 10.1016/j.neuron.2016.03.018 27100195 PMC4840471

[B30] Mitchell J. F. Reynolds J. H. Miller C. T. (2014). Active vision in marmosets: A model system for visual neuroscience. *J. Neurosci.* 34 1183–1194. 10.1523/JNEUROSCI.3899-13.2014 24453311 PMC3898283

[B31] Narayanan N. S. Laubach M. (2009). Delay activity in rodent frontal cortex during a simple reaction time task. *J. Neurophysiol.* 101 2859–2871. 10.1152/jn.90615.2008 19339463 PMC4280159

[B32] Pandey S. Simhadri S. Zhou Y. (2020). Rapid head movements in common marmoset monkeys. *iScience* 23:100837. 10.1016/j.isci.2020.100837 32058952 PMC6997856

[B33] Paré M. Munoz D. P. (2001). Expression of a re-centering bias in saccade regulation by superior colliculus neurons. *Exp. Brain Res.* 137 354–368. 10.1007/s002210000647 11355382

[B34] Peirce J. Gray J. R. Simpson S. MacAskill M. Höchenberger R. Sogo H. (2019). PsychoPy2: Experiments in behavior made easy. *Behav. Res. Methods* 51 195–203. 10.3758/s13428-018-01193-y 30734206 PMC6420413

[B35] Potier S. (2023). *Do birds have faster reaction times than humans?.* Available online at: https://www.newscientist.com/lastword/mg25734333-100-do-birds-have-faster-reaction-times-than-humans/ (accessed July 12, 2024).

[B36] Roll A. Wierzbicka M. M. Wolf W. (1996). The “gap paradigm” leads to express-like saccadic reaction times in Parkinson’s disease. *Exp. Brain Res.* 111:9562. 10.1007/BF00229562 8891643

[B37] Saslow M. G. (1967). Effects of components of displacement-step stimuli upon latency for saccadic eye movement. *J. Opt. Soc. Am.* 57 1024–1029. 10.1364/JOSA.57.001024 6035296

[B38] Schmidt R. Leventhal D. K. Mallet N. Chen F. Berke J. D. (2013). Canceling actions involves a race between basal ganglia pathways. *Nat. Neurosci.* 16 1118–1124. 10.1038/nn.3456 23852117 PMC3733500

[B39] Schmolesky M. T. Wang Y. Hanes D. P. Thompson K. G. Leutgeb S. Schall J. D. (1998). signal timing across the macaque visual system. *J. Neurophysiol.* 79 3272–3278. 10.1152/jn.1998.79.6.3272 9636126

[B40] Sedaghat-Nejad E. Herzfeld D. J. Hage P. Karbasi K. Palin T. Wang X. (2019). Behavioral training of marmosets and electrophysiological recording from the cerebellum. *J. Neurophysiol.* 122 1502–1517. 10.1152/jn.00389.2019 31389752 PMC6843097

[B41] Selvanayagam J. Johnston K. D. Everling S. (2024). Laminar dynamics of target selection in the posterior parietal cortex of the common marmoset. *J. Neurosci.* 44:e1583232024. 10.1523/JNEUROSCI.1583-23.2024 38627088 PMC11112649

[B42] Senn W. Dold D. Kungl A. F. Ellenberger B. Jordan J. Bengio Y. (2023). A neuronal least-action principle for real-time learning in cortical circuits. *bioRxiv* [Preprint]. 10.1101/2023.03.25.534198 PMC11661794 39704647

[B43] Solomon S. G. Rosa M. G. P. (2014). A simpler primate brain: The visual system of the marmoset monkey. *Front. Neural Circuits* 8:96. 10.3389/fncir.2014.00096 25152716 PMC4126041

[B44] Spinelli S. Pennanen L. Dettling A. C. Feldon J. Higgins G. A. Pryce C. R. (2004). Performance of the marmoset monkey on computerized tasks of attention and working memory. *Cogn. Brain Res.* 19 123–137. 10.1016/j.cogbrainres.2003.11.007 15019709

[B45] Srivastava A. Sharma R. Sood S. Shukla G. Goyal V. Behari M. (2014). Saccadic eye movements in Parkinson’s disease. *Indian J. Ophthalmol.* 62 538. 10.4103/0301-4738.133482 24881597 PMC4065501

[B46] Thepass G. Lemij H. G. Vermeer K. A. van der Steen J. Pel J. J. M. (2021). Slowed saccadic reaction times in seemingly normal parts of glaucomatous visual fields. *Front. Med.* 8:679297. 10.3389/fmed.2021.679297 34513866 PMC8426641

[B47] Tomasik B. (2016). *Do smaller animals have faster subjective experiences?* Available online at: https://reducing-suffering.org/small-animals-clock-speed/ (accessed July 12, 2024).

[B48] van Koningsbruggen M. G. Pender T. Machado L. Rafal R. D. (2009). Impaired control of the oculomotor reflexes in Parkinson’s disease. *Neuropsychologia* 47 2909–2915. 10.1016/j.neuropsychologia.2009.06.018 19560476 PMC2778793

[B49] Walker J. MacLean J. Hatsopoulos N. G. (2017). The marmoset as a model system for studying voluntary motor control. *Dev. Neurobiol.* 77 273–285. 10.1002/dneu.22461 27739220

[B50] Wei W. Wang X.-J. (2016). Inhibitory control in the cortico-basal Ganglia-thalamocortical loop: Complex regulation and interplay with memory and decision processes. *Neuron* 92 1093–1105. 10.1016/j.neuron.2016.10.031 27866799 PMC5193098

[B51] Yu H.-H. Chaplin T. A. Rosa M. G. P. (2015). Representation of central and peripheral vision in the primate cerebral cortex: Insights from studies of the marmoset brain. *Neurosci. Res.* 93 47–61. 10.1016/j.neures.2014.09.004 25242578

