# Supplementary figures and images for: Modeling saccade reaction time in marmosets: the contribution of earlier visual response and variable inhibition
Source: Front Syst Neurosci. 2024 Oct 23;18:1478019. doi: 10.3389/fnsys.2024.1478019 (PMC11537947; doi:10.3389/fnsys.2024.1478019)

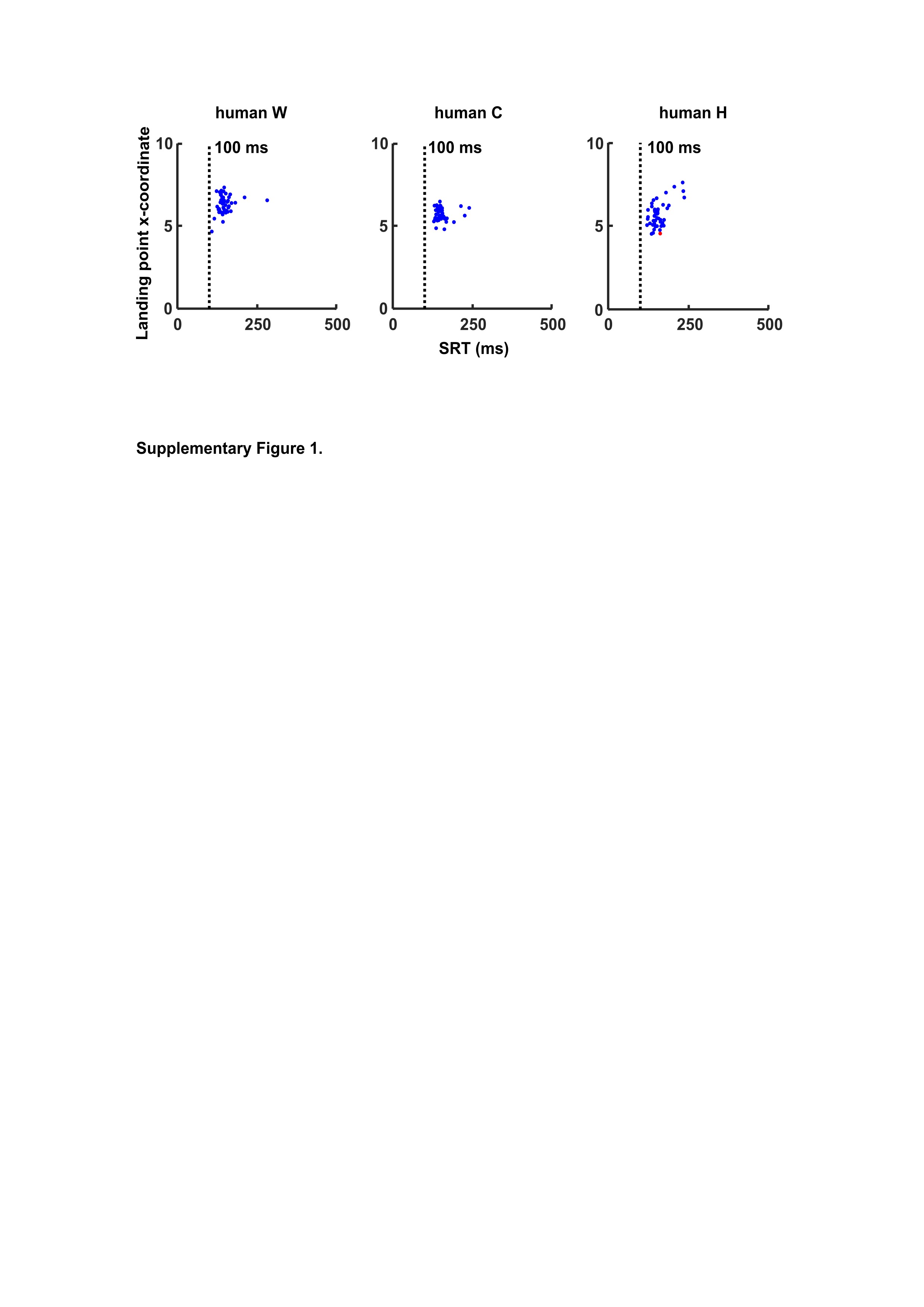

Supplement: Supplementary Figure 1 — Identification of anticipatory saccade thresholds in humans. Each scatterplot represents saccades made toward the rightward target. The landing point x-coordinate is plotted against primary saccade latency measured from the onset of the visual target at 6 deg eccentricity. Correct saccades are presented in blue and errant saccades are in red. No early errant saccades were made by human subjects. [file Image_1.JPEG]

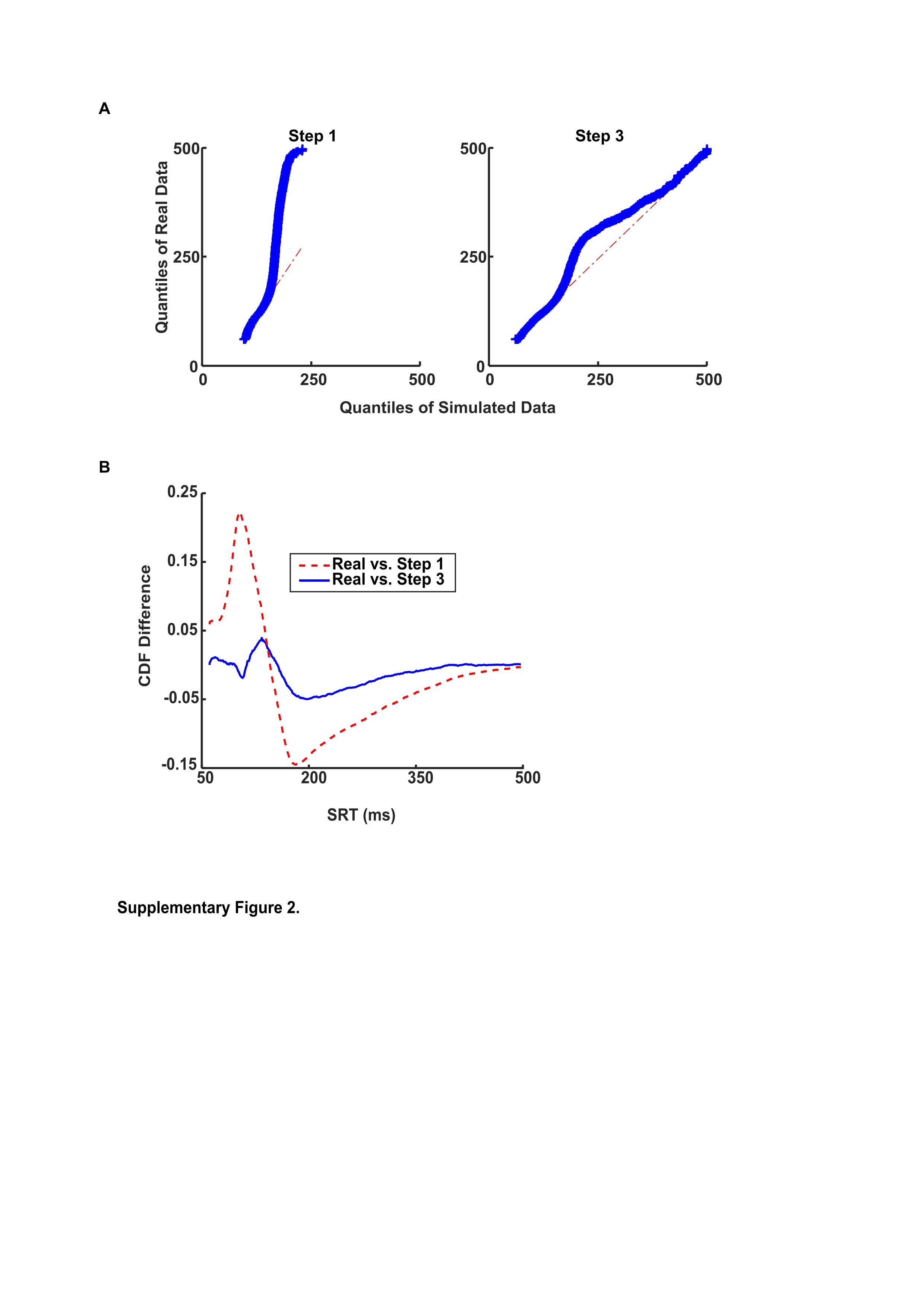

Supplement: Supplementary Figure 2 — Statistical figures demonstrating the superiority of step 3 modifications over step 1. (A) The Q-Q plot compares the quantiles of the simulated data to the real marmoset SRTs, assessing the fit and identifying deviations. In step 1, points in the Q-Q plot deviate from the straight line, indicating differences between the distributions. In contrast, the points in step 3 fall approximately along a straight line, suggesting that the distributions are more closely aligned. (B) Differences in the cumulative distribution functions between the real marmoset data and the simulated models were also evaluated. A smaller difference in CDFs indicates a better fit, with the step 3 model more effectively replicating the real marmoset SRT distribution than step 1. [file Image_2.JPEG]

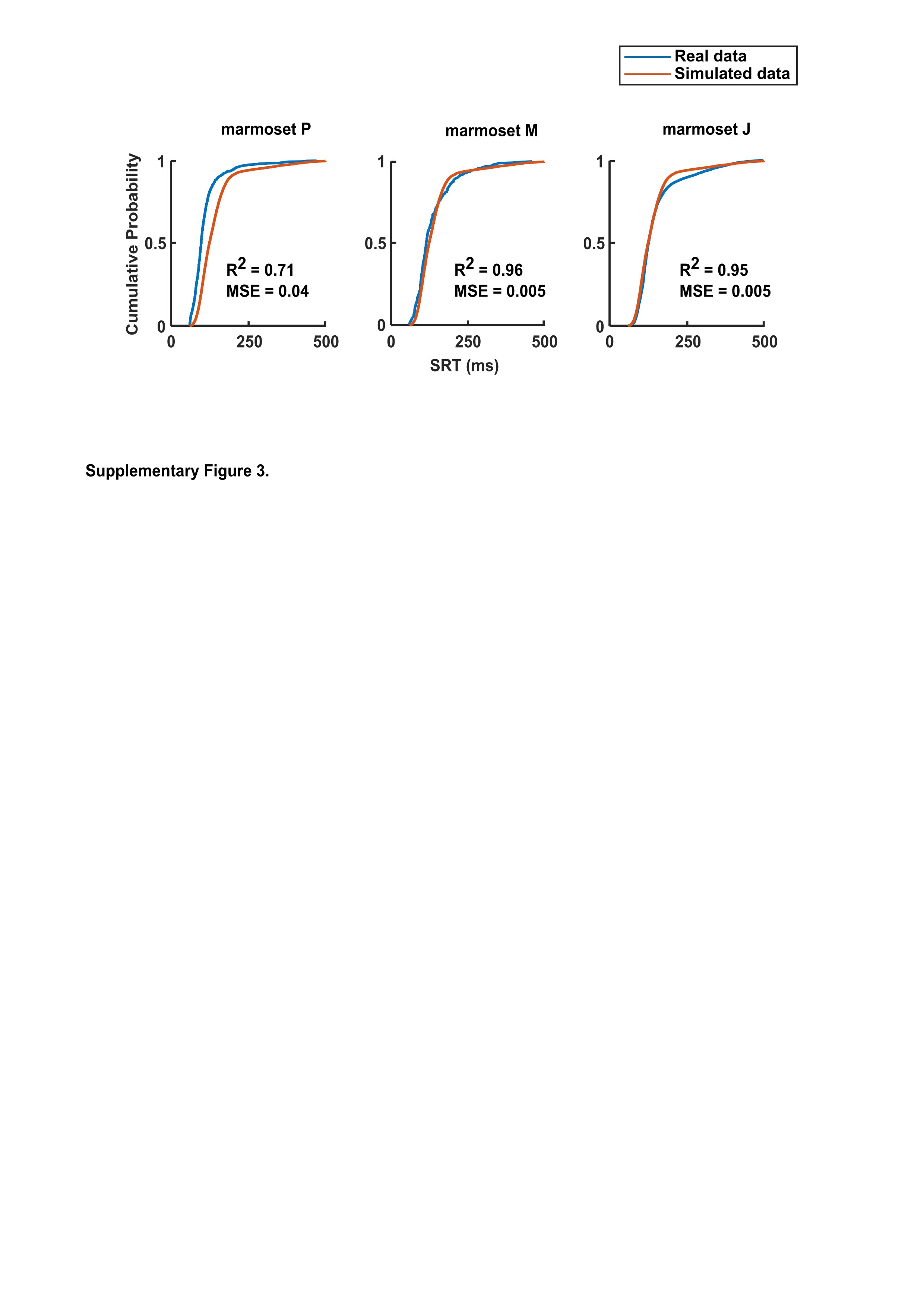

Supplement: Supplementary Figure 3 — Cumulative distribution of individual Marmoset SRTs and our Model. The figure illustrates how closely the model captures the overall SRT distribution of individual marmosets. [file Image_3.JPEG]
